# Bioenergetic signatures of circulating blood cells as biosensors of metabolic health in food-producing animals

**DOI:** 10.3389/fvets.2025.1653669

**Published:** 2025-10-08

**Authors:** Ignacio R. Ipharraguerre, Lorenzo A. Rosales Cavaglieri, Gerald Rimbach

**Affiliations:** Division of Food Science, Faculty of Agricultural and Nutritional Sciences, Institute of Human Nutrition and Food Science, University of Kiel, Kiel, Germany

**Keywords:** bioenergetics, mitochondrial function, circulating blood cells, respirometry, extracellular flux analysis, PBMCs, platelets, animal production

## Abstract

The intensification of animal production has substantially increased productivity, yet it has concurrently increased the metabolic vulnerability of livestock and poultry. Traditional biomarkers often lack sensitivity and fail to capture early or systemic dysfunction. In recent years, the bioenergetic profiling of circulating blood cells, particularly peripheral blood mononuclear cells and platelets, has emerged as a minimally invasive tool to assess mitochondrial function and systemic metabolic health. This critical review explores the application of blood-based bioenergetic assessments in food-producing animals, drawing parallels from human clinical research where such approaches have contributed to describe metabolic, inflammatory, and immune-related disorders. We highlight how respirometry and extracellular flux analysis enable high-resolution characterization of cellular respiration, glycolysis, and metabolic flexibility in circulating cells. Evidence from cattle, swine, and poultry suggests that circulating cell bioenergetics reflects both immune function and whole-body metabolic competence. We propose that this approach holds promise as a diagnostic and research tool to monitor physiological stress, support early intervention, and generate insights into breeding or nutritional strategies in food-producing animals. Finally, we identify key methodological and translational gaps that must be addressed to realize the full potential of this approach in animal production systems.

## 1 Introduction

The intensification of animal agriculture has led to remarkable gains in productivity across livestock and poultry species partly through genetic selection for traits like rapid growth, prolific reproduction, and high milk yield. This progress, however, has also introduced new physiological challenges. Modern genotypes, while highly productive, are increasingly prone to metabolic dysfunctions, particularly during physiologically demanding periods such as peripartum transition, early lactation, or rapid muscle accretion (1–3). These metabolic disorders are frequently linked to disruptions in energy homeostasis, oxidative stress, and persistent inflammation, suggesting an inherent but latent vulnerability embedded in the metabolic adaptability of high-producing animals.

According to the reallocation theory, the evolutionary prioritization of production traits has occurred at the expense of immune competence, fostering tolerogenic immune profiles and heightening the risk of chronic, unresolved inflammation (4). Whether or not the reallocation theory fully explains the metabolic vulnerability of modern livestock, an emerging paradox is evident: the same metabolic flexibility that enables high performance may also render animals more susceptible to homeostatic failure. Adaptive physiological responses, such as shifts in insulin sensitivity and nutrient partitioning during lactation, are essential for meeting elevated metabolic demands. However, under conditions of immune activation, nutrient imbalance, or sustained inflammation, this flexibility may become a liability (5, 6). Precisely because these mechanisms are adjustable, they are also more prone to dysregulation, ultimately leading to functional decline in key metabolic tissues. Therefore, new insights are needed to detect and monitor these early maladaptive transitions and to better understand the underlying mechanisms of metabolic failure. Blood metabolite panels, body condition scoring, or other sporadic clinical assessments often fall short in sensitivity, timeliness, and/or specificity (2, 7, 8). There is thus a pressing need for minimally invasive, sensitive, and mechanistically informative approaches that can identify vulnerable animals before the onset of overt disease. Such tools would not only enhance early detection and intervention but also illuminate the physiological processes that lead to metabolic dysfunction.

In this context, the emerging field of circulating cell bioenergetics provides a promising alternative. Drawing from advances in human medicine, the functional profiling of peripheral blood mononuclear cells (PBMCs) and platelets has proven to be a powerful, minimally invasive method to assess mitochondrial function and predict susceptibility to metabolic and inflammatory disorders (9, 10). These circulating cells reflect the organism's systemic bioenergetic and immune status, offering a dynamic “liquid biopsy” that can capture changes in mitochondrial respiration, adenosine triphostate (ATP) production, glycolytic capacity, and spare respiratory capacity, parameters that often shift prior to overt physiological dysfunction (11). Technologies such as high-resolution respirometry and extracellular flux (ECF) analysis allow the generation of bioenergetic signatures that characterize the energetic phenotype of blood cells under basal, stressed, or both conditions. In humans, these signatures have already been correlated with health disorders such as obesity, diabetes, and chronic fatigue. Importantly, they also offer insights into the mechanisms by which inflammation, oxidative stress, or nutrient stress modulate systemic metabolism (9–11).

Building on findings in human health research, our group and others have recently adapted bioenergetic profiling techniques for application in livestock and poultry species. While current applications in animal research have primarily focused on immune function (12–19), this is to our knowledge the first report to comprehensively evaluate circulating cell bioenergetics as an integrative platform for assessing metabolic function in food-producing animals. This review bridges the translational gap between human and animal research, synthesizes recent experimental applications in pigs, dairy cattle, and poultry, and outlines the conceptual basis for using blood cell bioenergetic signatures as real-time indicators of systemic metabolic stress. In doing so, it highlights both the mechanistic insights and practical diagnostic value of this emerging approach, while identifying key knowledge gaps and methodological priorities for future research. Ultimately, this review seeks to position circulating cell bioenergetic signatures as a versatile biomarker platform that not only supports early detection of metabolic dysfunction but also deepens our understanding of the energetic and immunometabolic underpinnings of animal performance and health.

## 2 Method

This article constitutes a critical review within the typology proposed by Grant and Booth (20). In line with he SALSA (Search, Appraisal, Synthesis, Analysis) framework, we identified relevant publications through targeted searches in Google Scholar, PubMed, and Scholar GPT. No formal quality appraisal was undertaken; instead, emphasis was placed on the conceptual contribution of each study. The selected literature was synthesized using a narrative, conceptual approach to integrate findings and frame new perspectives on circulating cell bioenergetics in food-producing animals.

## 3 High productivity at the edge of metabolic vulnerability

Over recent decades, genetic selection, nutritional strategies, and management practices have markedly increased productivity in livestock and poultry species. Such an increase in productivity, however, has not been without cost. A large body of evidence demonstrates a temporal association between rising productivity and the increased incidence of metabolic, inflammatory, and reproductive disorders (21–24). This trend suggests that although high-performing animals are metabolically equipped to support growing productivity, they operate increasingly close to the threshold of their metabolic resilience.

High productivity is granted by metabolic flexibility, which is the capacity of tissues to shift between energy substrates and reallocate nutrients in response to changing physiological demands (24). This flexibility is especially critical during the periparturient period, when lactogenesis begins and nutrient requirements rise sharply. In both dairy cows and sows, these adaptations involve coordinated lipolysis, hepatic gluconeogenesis, and glucose sparing for the mammary gland (6, 25). Compelling evidence indicates, however, that high-producing animals in modern production settings function increasingly close to the limits of their homeostatic capacity. For instance, as milk yield in dairy cows has steadily increased, doubling in many developed countries since the 1980s (26, 27), the prevalence of metabolic disorders such as subclinical ketosis, fatty liver, displaced abomasum, and hypocalcemia has also risen, particularly during early lactation (28, 29). Antagonistic genetic correlations clearly link higher milk yield with increased risk of metabolic disease and reduced fertility (24–27). Furthermore, despite decades of intensive research on the transition period, the prevalence of metabolic disorders in dairy cows remains persistently high (28). As reviewed by those authors, rates of subclinical ketosis typically range from 20 to 40%, while displaced abomasum, hypocalcemia, and retained placenta each occur in 5%−10% of cows, even in well-managed herds. A parallel pattern is observed in sows, where selection for hyperprolificacy has substantially increased the metabolic burden of late gestation and lactation. Over the last two decades, average litter sizes have increased from ~10 to 12 to over 14 piglets per parity, and the number of piglets weaned per sow per year has risen from around 20 to more than 30 in many farms worldwide (6, 25, 26). Although specific assessments of metabolic flexibility in peripartum sows are still lacking, surrogate indicators, such as postpartum dysgalactia syndrome (PPDS) (2) and rising sow mortality (23, 27, 28), suggest a similarly compromised metabolic state. Notably, annual sow mortality has nearly doubled over the past decade, now ranging between 8 and 15% in commercial settings in the US and Europe, with reproductive failure, locomotion impairment, and peripartum collapse (sudden death) among the most frequent causes (23, 27, 28).

In both dairy cows and sows, mounting evidence indicates that systemic inflammation is not only a transient response during parturition but often persists into early lactation and beyond. For instance, a recent survey involving 72 high-producing dairy herds in the US uncovered that 30%−40% of the postpartum cows exhibit signs of low-grade inflammation even in the absence of overt clinical disease (29). Similarly, in sows, parturition is accompanied by pronounced increases in circulating and salivary markers of stress and inflammation, including adenosine deaminase isoenzymes, haptoglobin, and cortisol (30). These responses frequently become pathological by extending beyond the immediate postpartum period, and failure to resolve inflammation rapidly has been linked to reproductive disorders such as PPDS, which continues to affect a significant proportion of hyperprolific sows despite advances in housing and management (2).

The inability of modern dairy cows to resolve inflammation shortly after parturition has promoted the view that metabolic vulnerability in high-producing animals may not merely be a downstream consequence of energy imbalance. The proposal by Horst et al. (5), along with recent findings from the same group (31), points to immune activation as the trigger of metabolic dysfunction, rather than an outcome of negative energy balance. According to these researchers, inflammatory insults during the peripartum period, such as mastitis, metritis, or endotoxemia, can impair metabolic flexibility by disrupting fuel partitioning and mitochondrial function. Immune-activated animals exhibit reduced fatty acid oxidation and increased reliance on lactate and glucogenic amino acids, ultimately limiting glucose availability for the mammary gland and compromising milk production. In this context, Horst et al. (5) further suggest that classical biomarkers like non-esterified fatty acids and ketone bodies may indeed be secondary consequences of an upstream immune-metabolic imbalance driven by inflammation-induced hypophagia, altered insulin dynamics, and competition for glucose by activated leukocytes. Whether immune activation is the primary causal factor or a contributing stressor, these observations reinforce the suggestion that tight coupling between metabolic throughput and performance leaves limited room for deviations so even modest perturbations may precipitate systemic imbalance, functional decline, and ultimately, the impairment of homeostatic regulation.

As the improvements in productivity continue to push the metabolic capacity of modern food-producing animals, the ability to detect early signs of physiological imbalance becomes increasingly important. In this context, functional biomarkers that capture real-time shifts in energy metabolism offer a clear advantage over conventional static measures. Circulating cell bioenergetics represents a promising approach not only to identify animals at risk before clinical dysfunction occurs but also to elucidate the underlying metabolic and mitochondrial mechanisms that dictate resilience or failure under stress.

## 4 Cellular bioenergetics for monitoring metabolic health

### 4.1 Principles of cellular bioenergetics

**Figure 1:**
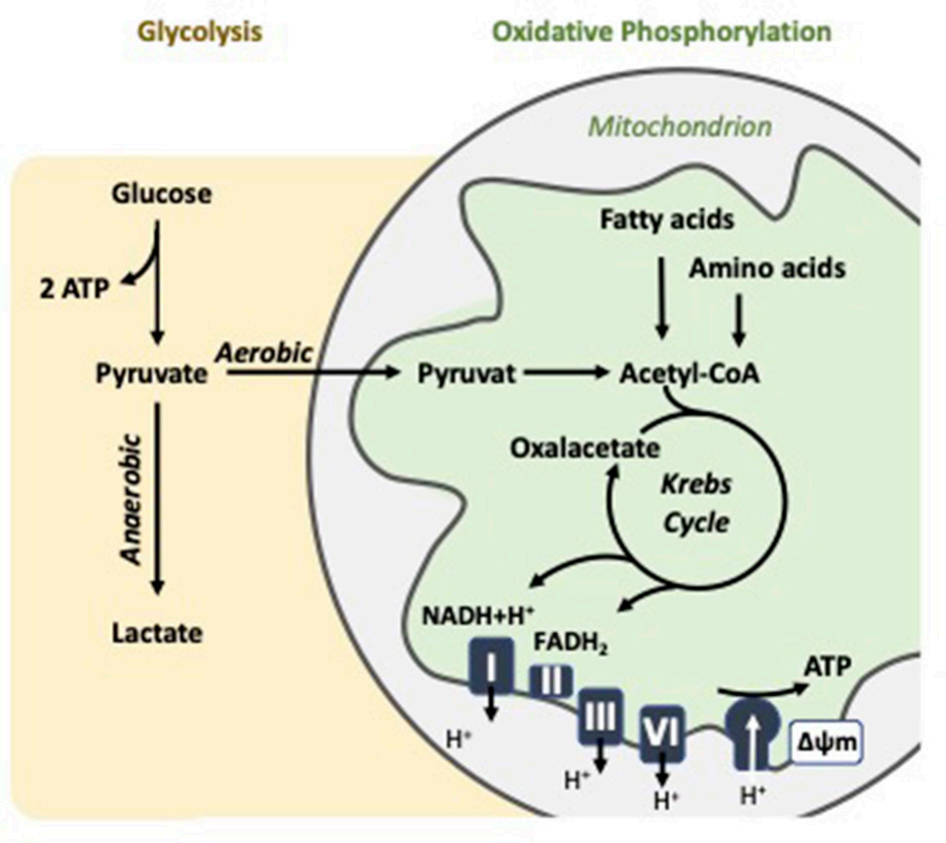
Cellular energy metabolism: Key features of oxidative phosphorylation and aerobic glycolysis in the mitochondrion.

Bioenergetics is concerned with the flow and transformation of energy within biological systems. At the core of this process is the mitochondrion, a dynamic organelle central to cellular metabolism. Mitochondria generate ATP, coupling the oxidation of nutrients like glucose, fatty acids, and amino acids with the production of cellular energy (Figure 1). The efficiency of this process is critical not only for energy supply but also for maintaining homeostasis, supporting cell proliferation, and modulating apoptosis, among other vital functions (32). Mitochondrial ATP production primarily occurs through oxidative phosphorylation (OXPHOS), which takes place in the inner mitochondrial membrane and involves the electron transport chain (ETC) and ATP synthase. This pathway is highly efficient, yielding up to about 36 ATP per glucose molecule, depending on the shuttle systems and conditions (32). However, cells can also generate ATP through glycolysis, a cytoplasmic process that converts glucose into pyruvate while producing a net gain of 2 ATP per glucose molecule. Under aerobic conditions, pyruvate typically enters mitochondria for oxidation via the Krebs cycle.

In some contexts, however, especially in rapidly proliferating cells like activated immune cells, pyruvate is preferentially converted to lactate even in the presence of oxygen. This phenomenon is known as aerobic glycolysis or the Warburg effect (33). Although less efficient in terms of ATP yield, aerobic glycolysis provides rapid energy and intermediates for biosynthetic pathways, offering selective advantages under conditions of high metabolic demand. The balance between OXPHOS and glycolysis varies by cell type, physiological condition, and pathological state. For instance, quiescent cells like resting lymphocytes and platelets predominantly rely on OXPHOS, whereas activated T cells and macrophages cells often shift toward aerobic glycolysis to support growth and proliferation (9, 34). This metabolic plasticity underscores the importance of measuring both mitochondrial respiration and glycolytic activity to understand cellular metabolism comprehensively.

### 4.2 Approaches to measure bioenergetic function in circulating blood cells

While early studies assessed mitochondrial activity through isolated enzyme assays in blood cells, such static methods lacked functional relevance and translational utility (32). As a result, bioenergetics research has shifted toward integrated, real-time assessments of mitochondrial function using respirometry platforms (35).

In this field, the Oroboros O2k high-resolution respirometry system (Oroboros Instruments, Austria) has become a gold standard for detailed mitochondrial analysis in intact cells and permeabilized preparations. This system provides highly sensitive, continuous measurement of oxygen flux in closed chambers, allowing for detailed evaluation of individual respiratory states under tightly controlled substrate and inhibitor conditions (36). It offers flexibility to assess mitochondrial coupling, substrate utilization, and complex-specific dysfunction, especially in settings where precision and mechanistic dissection are prioritized (37). In addition, several studies have successfully used Oroboros-based protocols to examine mitochondrial function in PBMCs and platelets from humans (38–40). More recently, the Seahorse XF Analyzer (Agilent Technologies) has emerged as a high-throughput platform for ECF analysis, enabling label-free, real-time quantification of oxygen consumption rate (OCR) and extracellular acidification rate (ECAR) in live cells. OCR serves as a proxy for mitochondrial respiration and ATP synthesis, while ECAR reflects glycolytic activity through lactate-associated proton efflux (41, 42). The system is particularly well-suited for monitoring dynamic metabolic changes in multiple cell populations under microplate conditions, requiring minimal sample volumes which is an advantage when working with peripheral blood cells (35). Consequently, both platforms provide complementary insights, with Seahorse excelling in functional screening and scalability, and Oroboros offering mechanistic depth and kinetic resolution.

**Figure 2:**
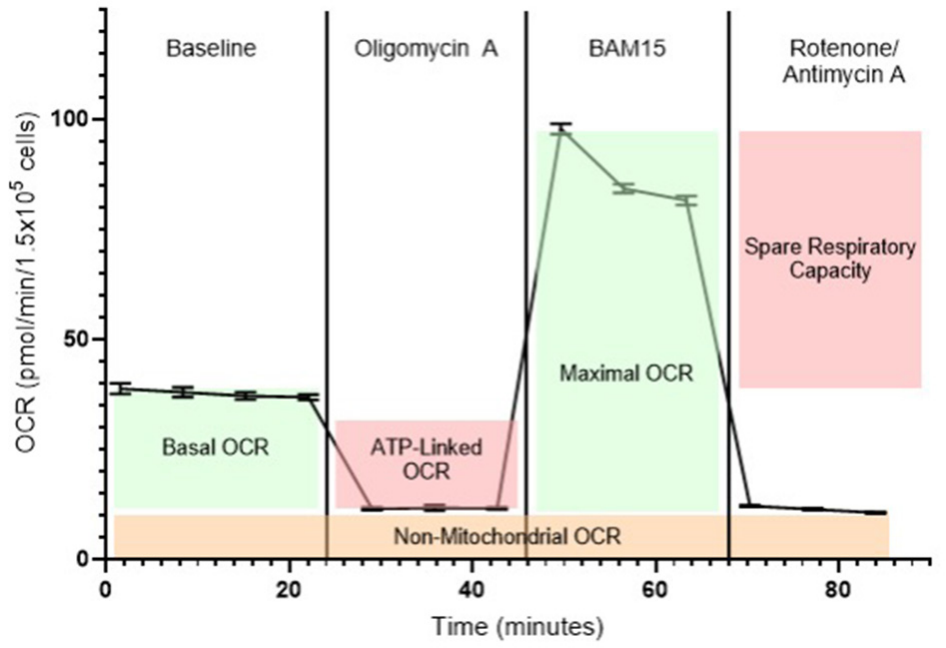
Representative bioenergetic profile of blood cells measured using the Seahorse XF analyzer. Baseline oxygen consumption rate (OCR) is recorded prior to any compound injections and reflects mitochondrial respiration under normal, unstimulated conditions. The first injection is oligomycin A, an ATP synthase (complex V) inhibitor, which decreases OCR by blocking ATP-linked respiration, revealing the portion of mitochondrial activity dedicated to ATP production. The second injection is BAM15, a mitochondrial uncoupling agent that collapses the proton gradient across the inner mitochondrial membrane. This drives maximal electron flow through the electron transport chain (ETC), enabling the measurement of maximal respiratory capacity. The final injection is a combination of rotenone (a complex I inhibitor) and antimycin A (a complex III inhibitor), which together halt mitochondrial electron transport entirely. The remaining OCR after this step represents non-mitochondrial respiration, attributable to oxygen-consuming processes outside the mitochondria, such as activity of NADPH oxidases and other oxidoreductases.

A hallmark of the Seahorse XF system is the mitochondrial stress test (MST), which generates a detailed bioenergetic profile through the sequential injection of ETC inhibitors (Figure 2). Beyond mitochondrial respiration, the glycolysis stress test (GST) enables functional assessment of glycolytic capacity. This test quantifies ECAR-based parameters following the sequential addition of glucose, oligomycin, and 2-deoxyglucose (2-DG). Key readouts of the MST and GST are (10, 32, 42):

1. *Basal OCR:* Respiration under steady-state conditions controlled by proton re-entry through ATP synthase, endogenous proton leak, and substrate oxidation, reflecting maintenance energy expenditure.
2. *ATP-linked OCR:* The portion of oxygen consumption coupled to ATP production in the basal state, reduced after cellular exposure to oligomycin (an ATP synthase inhibitor).
3. *Proton leak:* Residual respiration post-oligomycin, indicating uncoupled oxygen use and potential membrane inefficiency.
4. *Maximal OCR:* Maximum respiratory capacity obtained after ETC uncoupling with the agent BAM15 or carbonycyanide-p-triflutomethoxyphenylhydrazone (FCCP), reflecting the cell's bioenergetic potential.
5. *Spare respiratory capacity (SRC):* The difference between maximal and basal OCR, representing the cell's ability to increase mitochondrial respiration in response to stress or elevated energy demand. SRC serves as an indicator of metabolic flexibility and resilience under challenging physiological conditions.
6. *Non-mitochondrial OCR:* Residual oxygen uses after ETC inhibition with rotenone and antimycin A, representing oxygen consumption by cytoplasmatic oxidases and related enzymes.
7. *ECAR:* An indicator of glycolytic activity, reflecting lactate production and proton efflux as a result of glucose metabolism in the cytoplasm.
8. *OCR/ECAR ratio:* A measure of the balance between mitochondrial respiration and glycolysis, providing insight into the cell's metabolic phenotype (oxidative vs. glycolytic preference) under specific conditions.
9. *Basal glycolysis:* The rate of ECAR increase upon glucose addition.
10. *Glycolytic capacity:* The maximal ECAR reached after mitochondrial ATP synthesis is blocked by oligomycin.
11. *Glycolytic reserve:* The difference between glycolytic capacity and basal glycolysis, indicating the cell's potential to upregulate glycolysis upon activation or stress.
12. *Non-glycolytic acidification:* Residual ECAR following 2-DG injection, reflecting proton production from non-glycolytic sources.

These metrics provide a comprehensive and dynamic analysis of cellular energy metabolism and mitochondrial function, enabling the determination of metabolic phenotypes, shifts in fuel sources, and detection of bioenergetic impairment under physiological or pathological conditions. When applied to circulating blood cells such as PBMCs and platelets, this approach offers a minimally invasive, repeatable, and scalable method for monitoring systemic metabolism in humans (9, 10, 41) and, by extension, in food-producing animals.

### 4.3 Metabolic diversity and mitochondrial functions in peripheral blood cells

Blood has long served as a cornerstone of clinical diagnostics, offering a readily accessible reservoir of biomarkers for physiological assessment, disease detection, and prognosis. This diagnostic value arises from blood's continuous circulation through every organ system, where it not only facilitates nutrient delivery and waste removal but also participates in the exchange of hormones, metabolites, and inflammatory mediators. Through this systemic interaction, blood becomes a powerful surrogate tissue capable of reflecting metabolic perturbations occurring throughout the body (10). In line with this concept, a growing body of research in humans has identified the mitochondrial function of circulating PBMCs and platelets as a promising biomarker of systemic bioenergetic health. These cells contain functional mitochondria, glycolytic enzymes, and redox systems adapted to their specific immune or hemostatic roles. Importantly, when isolated from peripheral blood, these cells retain their bioenergetic profile and can be analyzed with increasing precision using assays such as high-resolution respirometry and ECF analysis (9, 35). One of the primary advantages of this approach is its scalability and accessibility because peripheral blood can be drawn through minimally invasive procedures, even in vulnerable populations. PBMCs and platelets can then be rapidly isolated *ex vivo* using standard protocols. Critically, these cells do more than reflect steady-state metabolism, they are dynamically responsive to systemic stimuli (10). Constantly exposed to circulating oxygen gradients, stress signals, and nutritional and inflammatory cues, their mitochondria adapt by modulating respiration, shifting metabolic substrate preference, and altering production of reactive oxygen species (ROS) (43). Beyond energy production, mitochondria in blood cells play indispensable roles in signaling and cellular homeostasis. Among others, these include regulation of ROS, calcium signaling, and apoptosis (43, 44). For instance, elevated mitochondrial ROS (mtROS) and mitochondrial-mediated apoptosis have been implicated in key functional processes such as platelet activation (45), dendritic cell maturation (46), and lymphocyte activation (47). These findings underscore that mitochondria serve not only as metabolic engines but also as central regulators of immune activity, cellular differentiation, and systemic metabolic integration.

All circulating blood cells originate from hematopoietic stem cells in the bone marrow and differentiate into functionally distinct subsets along either myeloid or lymphoid lineages (48, 49). These include platelets, neutrophils, monocytes, and lymphocytes, each with unique metabolic profiles shaped by their developmental trajectories and specialized functions. PBMCs, a group comprising monocytes and lymphocytes, are distinguished by their round nuclei and are routinely isolated via Ficoll gradient centrifugation. In contrast, polymorphonuclear leukocytes (PMNs) such as neutrophils, and anucleate cells like platelets are not encompassed by the PBMCs classification (50). These distinctions correspond to important differences in mitochondrial content, metabolic flexibility, and bioenergetic profile, as briefly described below.

#### 4.3.1 Monocytes

Monocytes arise from the myeloid lineage and serve as circulating precursors to macrophages and dendritic cells. In a resting state, monocytes predominantly rely on OXPHOS, but they switch to aerobic glycolysis upon activation to support rapid cytokine production and ROS generation (9). This metabolic flexibility enables adaptation to inflammatory cues such as hyperglycemia or oxidized lipids, which are characteristic of metabolic diseases in humans. Mitochondria in monocytes play essential roles in immune signaling, especially through mtROS-mediated NLRP3 inflammasome activation and control of apoptotic thresholds (45). They also influence differentiation fates as mitochondrial membrane potential and substrate use shape whether monocytes polarize toward inflammatory (M1) or resolving (M2) macrophage states (51). These bioenergetic traits make monocytes a useful model for examining systemic immunometabolic health.

#### 4.3.2 Lymphocytes

Lymphocytes (T cells, B cells, and natural killer cells) arise from the lymphoid lineage and demonstrate remarkable metabolic plasticity. In humans, naïve and memory lymphocytes primarily depend on OXPHOS, while activated effector cells rapidly upregulate both glycolysis and mitochondrial respiration (9). Increased mitochondrial oxygen consumption during activation is tightly coupled to clonal expansion and cytokine secretion (47). Beyond bioenergetics, mitochondria regulate calcium signaling during antigen stimulation and control apoptosis via cytochrome c release and the Bcl-2 protein family (52). Because lymphocytes are both metabolically sensitive and immunologically central, their mitochondrial function is highly relevant for diseases marked by immune dysregulation.

#### 4.3.3 Neutrophils

Neutrophils are short-lived PMN cells of myeloid origin that rely almost entirely on glycolysis for ATP production. However, despite limited mitochondrial respiration, their mitochondria serve critical roles in apoptosis and ROS production (53). Mitochondrial signaling influences neutrophil activation, degranulation, and NET formation. While not suitable for studying OXPHOS, neutrophils offer a unique insight into redox shifts in acute inflammation and infectious disease.

#### 4.3.4 Platelets

Platelets are cytoplasmic fragments of megakaryocyte lineage, rich in mitochondria but devoid of nuclei. Human platelets use both glycolysis and OXPHOS to meet energy demands for clotting, immune signaling, and maintenance of resting potential (54, 55). They regulate inflammation by releasing respiratory-competent mitochondria that act as damage associated molecular patterns, promoting neutrophil recruitment and NETosis (56). Due to their lack of a nucleus, platelets cannot replenish mitochondrial proteins, making their mitochondrial health a highly sensitive measure of recent systemic stress. This feature, along with ease of access and high metabolic activity, has positioned platelets as an ideal platform for liquid biopsy assessment of systemic mitochondrial function in humans (11, 57).

### 4.4 Circulating blood cells as surrogates of tissue-specific mitochondrial function

One of the most clinically and biologically compelling aspects of blood cell bioenergetics is their ability to reflect mitochondrial function in solid tissues, even in metabolically specialized organs. While it may initially seem counterintuitive, given the diverse energetic demands and mitochondrial specialization across tissues, there is now substantial evidence supporting the concordance of bioenergetic parameters between peripheral blood cells and tissue-specific mitochondria. For example, Tyrrell et al. (57) demonstrated in non-human primates that monocyte and platelet bioenergetics, particularly basal OCR, maximal respiration, and SRC, correlated strongly with OXPHOS function in permeabilized skeletal muscle fibers from the same animals. These findings were extended to humans by Braganza et al. (58), who showed that platelet maximal OCR and proton leak correlated significantly with mitochondrial respiration in muscle biopsies, and ATP-linked OCR correlated with ATP production assessed non-invasively by P magnetic resonance spectroscopy in skeletal muscle. These studies provide strong support for using peripheral blood cells as viable surrogates for muscle mitochondrial function, avoiding the need for invasive biopsies.

It is important to note that the surrogate utility of blood cells extends beyond skeletal muscle. In the same primate study by Tyrrell et al. (57), platelet and monocyte OCR parameters were shown to correlate with respiratory control ratios in cardiac mitochondria. Similarly, monocyte maximal respiration mirrored mitochondrial function in the frontal cortex of the brain, a tissue with remarkably unique metabolic demands (59). In subsets of animals assessed with ^2^8F-fluorodeoxyglucose PET imaging, platelet maximal OCR was significantly associated with glucose uptake in diverse brain regions, denoting the ability of blood cell mitochondrial function to mirror metabolic activity in the central nervous system. Further supporting this translational value, Winnica et al. (60) demonstrated that in humans platelet bioenergetics reflected mitochondrial respiration and basal glycolytic rate in primary airway epithelial cells from the same individuals across lean, obese, and asthmatic cohorts. Importantly, while absolute bioenergetic values differ across tissues due to their specialized energetic needs; for example, brain mitochondrial maximal capacity being twice that of monocytes (59), relative differences and dynamic responses show meaningful agreement. This suggests that although tissue mitochondria are specialized, they remain sensitive to and influenced by systemic metabolic cues, which are captured in the circulating cell pool (10).

### 4.5 Blood cell bioenergetics in metabolic dysfunction

A growing body of literature supports the idea that in humans mitochondrial dysfunction is a hallmark of metabolic diseases, including obesity, type 2 diabetes, cardiovascular disease, chronic fatigue syndrome, and metabolic syndrome. These disorders are marked by disrupted OXPHOS, diminished ATP production, increased ROS generation, and impaired substrate switching. While traditionally attributed to dysfunction in individual tissues, such as skeletal muscle, liver, or pancreas, it is increasingly evident that mitochondrial dysfunction may be systemic in nature, affecting blood cells and other peripheral compartments in parallel with solid organs (9, 11, 61). Numerous studies now demonstrate that OCR, SRC, and glycolytic flux are significantly altered in blood cells from individuals with metabolic disorders. For instance, Wilkinson and Dunham-Snary (11) highlighted how mitochondrial rewiring in blood cells, particularly reduced glycolysis and altered glucose oxidation, mirrors bioenergetic adaptations to lipidemic stress and metabolic overload. Similar findings have been reported in patients with chronic fatigue syndrome, where PBMCs exhibit significantly reduced basal respiration, ATP production, and SRC, suggesting compromised mitochondrial function and cellular energy availability even in the absence of structural tissue damage (62). These findings underscore the potential for blood cell profiling to capture early, systemic mitochondrial dysfunction, even in the absence of overt organ failure.

Mechanistic insights into these changes have been detailed by Stefano et al. (63), who described how human hyperglycemia disrupts mitochondrial signaling in leukocytes, inducing oxidative stress, impairing mitochondrial plasticity, and disrupting redox balance. These effects may contribute to immune dysregulation and low-grade inflammation, both of which are central to the pathogenesis of type 2 diabetes and obesity. Platelets also exhibit profound bioenergetic shifts in metabolic disease. In patients with type 2 diabetes and metabolic syndrome, Kramer et al. (9) observed significant reductions in platelet maximal respiration and SRC, two parameters linked to mitochondrial efficiency and disease severity. Similarly, Cardenes et al. (64) demonstrated that platelets from individuals with sickle cell disease, a chronic inflammatory condition, exhibit metabolic remodeling consistent with increased oxidative stress and impaired ATP-linked respiration. Adding further clinical relevance in humans, Morton et al. (65) observed distinct bioenergetic signatures among leukocyte subtypes in patients with acute pancreatitis, reinforcing the idea that mitochondrial stress is both cell-type specific and reflective of broader systemic dysfunction.

Taken together, these findings underscore that in humans circulating blood cells are not passive indicators but active reporters of systemic mitochondrial health, capable of capturing pathogenic changes that are otherwise difficult to detect. As such, blood-based bioenergetic profiling not only enables early detection and monitoring but also offers a unique opportunity to interrogate the molecular mechanisms underlying metabolic disease progression.

## 5 Blood cell bioenergetics in food-producing animals

Although blood cell bioenergetics has gained significant attention in human translational research, its application in animal production is just beginning to expand. In this field, mitochondrial function is increasingly being recognized as a critical determinant of metabolic efficiency, health, and performance. In cattle, for instance, pioneering studies such as those by Kolath et al. (66) reported reduced mitochondrial function in both muscle and blood cells of low-efficiency Angus steers, suggesting that bioenergetic inefficiency is systemically expressed and detectable in peripheral tissues. Extending these findings, Bottje and Carstens (67) linked mitochondrial respiration to feed efficiency, showing that animals with low residual feed intake (RFI) exhibit enhanced mitochondrial function and reduced oxidative stress, not only in muscle but also in blood-derived cells. In Nellore bulls, Baldassini et al. (68) further demonstrated that blood mitochondrial DNA copy number and heat production correlated with RFI, adding a genomic layer to bioenergetic profiling in livestock. Despite their value, these initial efforts relied heavily on traditional biochemical techniques, which required isolation of mitochondria from tissue biopsies or large blood volumes, and primarily focused on static markers such as mitochondrial DNA content, enzyme activity, or redox status (66, 68). While these methods laid the groundwork for understanding mitochondrial dynamics in food-producing animals, they were limited in sensitivity, scalability, and physiological relevance, especially for real-time metabolic analysis in living cells. These shortcomings are now overcome by the advent of ECF analysis, which (as outlined below) is rapidly emerging as a novel approach for investigating blood cell bioenergetics in farm animals.

In dairy cattle, Eder et al. (15) pioneered the use of ECF analysis to profile CD4? T cells across lactation stages. Their results demonstrated that cows in late lactation or the dry period had greater mitochondrial mass and glycolytic potential than those in early lactation, identifying lactation stage as a modulator of immune metabolism and metabolic vulnerability. Building on this, Kesler and Abuelo (19) investigated the mitochondrial function of lymphocytes in dairy calves from birth to immunologic maturity and found that, while early postnatal differences existed, lymphocyte OXPHOS capacity remained relatively stable over time, suggesting that developmental stage has limited impact on lymphocyte mitochondrial function in dairy cattle. Most recently, Arshad et al. (16) developed a ECF assay to analyze CD4? T cell bioenergetics in lactating cows and demonstrated that immune activation induces a glycolytic switch, mirroring immunometabolic remodeling patterns observed in humans. In follow-up work, they compared high- and low-efficiency dairy cows and found that efficient animals displayed superior mitochondrial and glycolytic activity in CD4? T cells, indicating enhanced immunometabolic flexibility associated with feed efficiency (17).

In poultry, Meyer et al. (18) optimized and applied ECF protocols to measure OCR and ECAR in chicken PBMCs across genetic lines. Their study found that vaccinated birds shifted toward mitochondrial ATP production, with layer-type birds demonstrating higher metabolic rates than broilers. Interestingly, legacy lines had superior baseline mitochondrial function compared to modern commercial lines, suggesting that intensive genetic selection for production traits may have compromised immunometabolic fitness (18).

**Figure 3:**
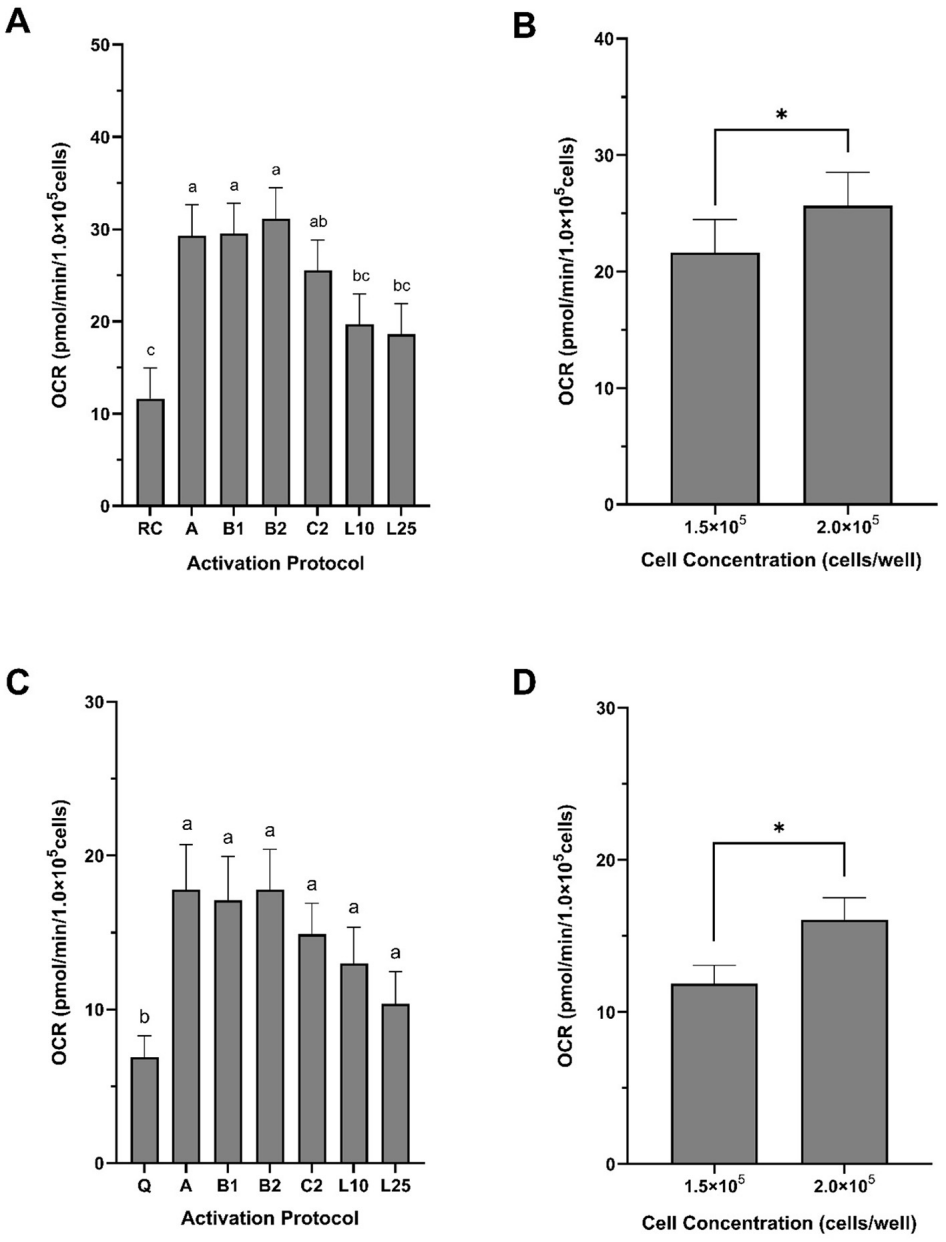
Maximal oxygen consumption rate and spare respiratory capacity of peripheral blood mononuclear cells (PBMCs) isolated from growing pigs. PBMCs were isolated from whole blood collected from healthy, 3-month-old female pigs. **(A, B)** Maximal oxygen consumption rate (OCR) and **(C, D)** spare respiratory capacity (SRC) are expressed in pmol/min/1 × 10^5^ cells. Both bioenergetic parameters were significantly affected by activation protocol **(A, C)** and cell concentration **(B, D)**. Activation protocols were: A = 20 ng/ml PMA + 1 μg/ml ION; B1 = 40 ng/ml PMA + 1 μg/ml ION; B2 = 200 ng/ml PMA + 1 μg/ml ION; C2 = 20 ng/ml PMA + 10 μg/ml ION; L10 = 10 μg/ml LPS; L25 = 25 μg/ml LPS. Data are presented as least squares means ± SEM in panels **A** and **B**, and as arithmetic means ± SEM in panels **C** and **D** (*n* = 5), normalized to 1 × 10^5^ cells/well. In panel **A**, bars with different superscript letters^a,b,c^ indicate significant differences due to activation protocol. In panels **C** and **D**, asterisks (*) indicate a significant effect of cell concentration (*F*-test, *P* < 0.0001; χ^2^ test, *P* < 0.04). G1.5 = 1.5 × 10^5^ cells/well; G2.0 = 2.0 × 10^5^ cells/well.

In swine, recent studies validate the translational relevance of the ECF analysis for immunometabolic research. Giese et al. (12) examined PBMCs bioenergetics in INSC94Y transgenic pigs with chronic hyperglycemia and observed increased mitochondrial respiration and glycolytic activity. These shifts were associated with reduced lymphocyte proliferation and altered protein expression, suggesting systemic immune and metabolic dysregulation. In a related study, Ma et al. (13) evaluated the metabolic response of porcine PBMCs to inflammatory (lipopolysaccharide, LPS) and anti-inflammatory (dexamethasone) stimuli, demonstrating increased glycolytic flux with LPS and suppression of both glycolysis and OXPHOS with dexamethasone. Building on this foundation, our group has developed and validated a protocol for isolating and analyzing PBMCs bioenergetics in pigs using ECF technology under both quiescent and immunologically stimulated conditions (69). In preliminary experiments with healthy, growing pigs, we quantified several bioenergetic parameters following graded stimulation with phorbol 12-myristate 13-acetate (PMA), ionomycin (ION), and LPS. Among the activation protocols tested, PMA and ION combinations elicited the most robust and reproducible increases in mitochondrial respiration and glycolytic flux (data not shown). Importantly, maximal OCR and SRC were significantly influenced not only by stimulation but also by cell seeding density (Figure 3), underscoring the importance of assay optimization for ensuring reproducible outcomes.

These data represent an important first step toward establishing reference bioenergetic profiles for circulating blood cells in food-producing animals. Importantly, they align with the translational framework proposed by Braganza et al. (10), who emphasized the need for methodological standardization, the definition of healthy metabolic benchmarks, and quantification of intra- and inter-individual variability to support the diagnostic use of blood cell bioenergetics. In parallel, available evidence across animal species suggest that the bioenergetic profiling of circulating blood cells offers a robust and minimally invasive method for capturing systemic metabolic adaptations in response to physiological stress. When applied to high-producing animals, this approach has the potential to uncover early cellular signatures of metabolic vulnerability during demanding phases such as peripartum, lactation, and rapid growth.

## 6 Current limitations and future research priorities

As this review highlights, the functional profiling of circulating blood cells represents a novel and increasingly validated approach to assess mitochondrial and metabolic health in food-producing animals. Yet, despite promising advances, the wide implementation of blood-based bioenergetics in investigating metabolic function in farm animals, and its eventual translation into routine livestock health monitoring or decision-support tools, is still in its infancy (Table 1). To consolidate the utility of this approach as both a research platform and diagnostic tool, several key limitations must be addressed. In particular, we identify four high-priority research directions for the field:

1. Expansion of profiling protocols to include platelets and other blood cells.
2. Standardization of assays, reference ranges, and sample handling procedures.
3. Identification and control of potential confounding factors that modulate mitochondrial function in blood cells.
4. Mechanistic and translational validation of blood cells as surrogates for tissue-level metabolism.
5. Validation of bioenergetic markers as predictors of health and performance traits.

**Table 1:** Comparative evidence on circulating blood cell bioenergetics in humans and food-producing animals: key learnings and major knowledge gaps.

**Item**	**Humans**	**Food-producing animals**
Cell types studied	PBMCs, monocytes, lymphocytes (T, B, NK cells), neutrophils, platelets (9–11, 35)	Bovine: PBMCs, T cells, B cells (14, 16, 17, 19) Swine: PBMCs (12, 13, 69) Poultry: PBMCs (18)
Bioenergetic parameters measured	Full ECF readouts (9–11)	Bovine: MST and GST readouts (14, 16, 17, 19) Swine: MST readouts (12, 13, 69) Poultry: Basal and maximal OCR/ECAR (18)
Methodological status and readiness	Several standardized-validated protocols, partially validated cryopresevartion of blood cells, emerging reference ranges (9–11, 35, 70–73)	Bovine: early-stage protocols in T cells and PBMCs (14, 16, 17, 19) Swine: early-stage protocols in PBMCs Poultry: proof-of-concept assays in PBMCs (18)
Physiological and pathologicals contexts investigated	Metabolic disorders (obesity, type 2 diabetes), cardiovascular disease, chronic fatigue, sepsis, aging, inflammatory disorders, healthy individuals (9, 11, 61–64, 78)	Bovine: peripartum period, lactation, early-life immune development (calves), feed conversion efficiency (14, 16, 17, 19) Swine: growing phase, hyperglycemia (transgenic diabetic pigs), immune response (12, 13, 69) Poultry: genetic lines, immune response (vaccination) (18)
Correlation with tissue bioenergetics	Demostrated correlations with skeletal muscle, brain, cardiac tissue, respiratory epithelium (57–60)	Bovine. NA Swine: NA Poultry: NA
Correlation with health and performance outcomes	Established correlations with disease severity, prognosis, metabolic dysfunction (9, 11, 61–64)	Bovine: links with feed efficiency, immunometabolic shifts during lactation (14, 16, 17, 19) Swine: links with immunometabolic function during hyperglycemia, immune activation (12, 13, 69) Poultry: assoaciation with genetic selection for increased production (18)
Oustanding research needs	Limited cell-type specific standardization and preservation protocols, longitudinal studies, intervention studies controlling for confounding factors (10, 38, 79)	Across species expand protocols to platelets/other cells, standardize assays, establish reference ranges, assess confounding factors, validate blood–tissue links, test predictive value for systemic metabolism, health, performance

ECAR, extracellular acidification rate; ECF, extracelullar flux analysis; GST, glycolysis stress test; MST, mitchondrial stress test; NK, natural killer cells; OCR, oxygen consumption rate; PBMCs, peripheral blood mononuclear cells.

Current research efforts in livestock and poultry species have demonstrated the applicability of ECF analysis to PBMCs and T lymphocytes. To complement these efforts, there is a clear need to develop and validate protocols for platelet bioenergetic profiling in animal studies. Given their high mitochondrial content, systemic sensitivity, and ease of isolation, platelets may serve as a robust platform for monitoring metabolic health in farm animals.

Broader adoption and biological interpretation of blood cell bioenergetics will require the development of standardized reference ranges across physiological states, sex, and genetic backgrounds. Establishing what constitutes a “healthy” bioenergetic profile is fundamental to identifying deviations indicative of stress or dysfunction. In addition, challenges related to scalability must be addressed. Bioenergetic profiling, particularly via high-resolution respirometry and ECF approaches, requires sophisticated equipment, technical expertise, and strict assay standardization, factors that limit feasibility for large-scale or on-farm applications. Although several studies in humans have demonstrated partial success in preserving mitochondrial function following cryopreservation, particularly for PBMCs (70, 71), evidence also indicates that certain bioenergetic parameters are highly sensitive to freezing and handling conditions (72, 73). To date, no such preservation protocols have been validated for food-producing animals. Therefore, it is important to evaluate cryopreservation and short-term storage strategies to maintain bioenergetic integrity in farm animal PBMCs and platelets, enabling flexible sample handling for controlled, large-scale, or farm-based studies.

Another important but under-discussed limitation is the influence of confounding variables on mitochondrial function. Factors such as diet composition, microbiota, and environmental stress may independently modulate mitochondrial function in blood cells (74–77). These variables may mask or mimic underlying metabolic dysfunction, highlighting the need for controlled study designs and robust metadata collection to aid interpretation and improve translational accuracy.

A recurring theme throughout this review is the research focus on the interplay between mitochondrial metabolism and immune function. Notably, PBMCs and platelets are not merely passive indicators but active contributors to inflammation, redox balance, and metabolic adaptation. As such, their bioenergetic profiles offer valuable surrogate insights into systemic metabolic function and dysfunction. A key future direction involves leveraging these bioenergetic signatures to elucidate the molecular and cellular pathways through which metabolic flexibility becomes compromised in high-producing animals, particularly during physiologically demanding phases such as the peripartum period, lactation, or rapid postnatal growth. To this end, one of the most important yet underexplored priorities is the verification of circulating blood cells as valid surrogates for mitochondrial function in metabolically active tissues. The use of livestock models presents a unique opportunity in this regard, as invasive sampling of tissues such as liver, adipose, skeletal muscle, mammary gland, and gut is more feasible than in human research. Comparative studies that profile both circulating cells and target tissues within the same animals are needed to establish mechanistic correlations and validate the diagnostic relevance of blood-based bioenergetic signatures.

Beyond mechanistic insights, the direct association between bioenergetic parameters and performance outcomes in livestock remains limited. The predictive value of metrics of mitochondrial function for productivity, disease resistance, or reproductive efficiency in farm settings has yet to be rigorously validated, rendering their practical relevance more conceptual than operational. Without such validation, their integration into herd-level monitoring or decision-making frameworks will remain constrained. Therefore, longitudinal studies are especially needed to correlate changes in blood cell bioenergetics with subsequent health and performance traits like milk yield, reproductive success, or culling rate.

Although this review primarily focuses on pigs and dairy cattle, which are the species for which bioenergetic profiling of circulating blood cells has been most published, we acknowledge that small ruminants such as sheep and goats also play a critical role in global animal production. While not covered herein, many of the concepts and methodologies discussed are liklely transferable to these species and should be explored in future research to broaden our understanding of mitochondrial adaptability across diverse production systems.

In summary, circulating blood cell bioenergetics holds substantial promise for advancing our understanding of metabolic health and adaptability in food-producing animals. Its strengths, particularly minimally invasive sampling, cellular specificity, and mechanistic relevance, make it a powerful complement to existing research and diagnostic approaches. Yet, for this tool to evolve from a promising research platform into a practical component of progressive livestock management, current limitations in validation, scalability, confounding factors, and sample preservation must be overcome. At present, methodological complexity, cost, and throughput restrict its use to controlled laboratory settings. However, if these barriers can be addressed through technological innovation and assay standardization, bioenergetic profiling could be realistically integrated into herd/flock-level monitoring frameworks. However, if these barriers can be addressed through technological innovation and assay standardization, bioenergetic profiling could be realistically integrated into group-level monitoring frameworks. Some potential applications would include (1) early identification of animals at risk of metabolic dysfunction (e.g., during peripartum, peak lactation, or rapid growth phases), enabling targeted nutritional or management interventions; (2) surveillance of herd/flock metabolic status as part of precision animal farming, providing dynamic indicators of how groups of animals respond to feed changes, environmental stress, or disease challenges; (3) incorporation into breeding and selection programs, where bioenergetic parameters might serve as functional biomarkers of resilience, fertility, or feed efficiency; and (4) decision-support for veterinary and management practices, by helping to differentiate between primary metabolic dysfunction and secondary effects of confounding factors such as diet or infection. In this way, blood cell bioenergetics could ultimately enhance decision-making at the group level, complementing established health and performance monitoring tools and contributing to more proactive and sustainable animal management.
